# Essential updates 2022/2023: A review of current topics in robotic hepatectomy

**DOI:** 10.1002/ags3.12817

**Published:** 2024-06-21

**Authors:** Tomohiko Adachi, Takanobu Hara, Hajime Matsushima, Akihiko Soyama, Susumu Eguchi

**Affiliations:** ^1^ Department of Surgery Nagasaki University Graduate School of Biomedical Sciences Nagasaki Japan

**Keywords:** clinical trail, hilar cholangiocarcinoma, robotic hepatectomy, transplantation, vessel reconstruction

## Abstract

The liver requires careful handling intra‐operatively owing to its vital functions and complexity. Traditional open hepatectomy, while standard, is invasive and requires long recovery periods. Laparoscopic hepatectomy is a less invasive option, with its own challenges. The rise of robotic surgery, such as the da Vinci® system, improves precision and control, addressing the limitations of conventional methods, but brings new concerns, such as costs and training. This review focuses on the latest advancements in robotic hepatectomy from 2022/23 articles, delving into topics like “robotic surgery in liver transplantation,” “robotic hepatectomy for hilar cholangiocarcinoma,” “robotic vascular reconstruction following hepatectomy,” “robotic repeat hepatectomy,” and “prospective trials in robotic hepatectomy.” To retrieve articles, a focused literature search was conducted using PubMed for articles from 2022/23 with a 5‐year filter, excluding reviews. Initially, abstracts were screened, and relevant articles on robotic surgery were examined in full for inclusion in this review. Although all the above items are cutting‐edge, and many of the references are necessarily at the level of case reports, recent articles are still accompanied by surgical videos, which are useful to readers, especially surgeons who are considering imitating the procedures. In summary, we examined the recent advancements in robotic liver resection. The inclusion of videos that present new techniques aids in knowledge transfer. We anticipate the continued growth of this field of research.

## 1 INTRODUCTION

The liver is a vital organ that performs an array of essential physiological functions and demands meticulous attention and skill during surgical interventions to ensure the preservation of its complexity and capability. Hepatic resections, whether partial or not, necessitate a keen understanding of the hepatic anatomy and a deft surgical approach, particularly in scenarios involving tumor resections or transplants. Historically, open hepatectomy has been the standard approach despite being associated with notable morbidity and a prolonged recovery period owing to its invasive nature.^1^

In the pursuit of minimizing patient trauma and improving surgical outcomes, the advent of laparoscopic hepatectomy has introduced a minimally invasive alternative, offering reduced blood loss, shorter hospital stays, and less postoperative pain, albeit with its own set of challenges, including a steep learning curve for surgeons and limitations in precision.^2^

The dawn of the robotics era in surgical interventions has opened new avenues for addressing the challenges posed by traditional and laparoscopic methods. Robotic‐assisted hepatectomies employing platforms such as the da Vinci® Surgical System enhance the surgeon's dexterity and visualization through 3D magnified views and articulated instruments, offering a potential solution to the limitations of hand tremors and restricted movement encountered in conventional laparoscopic surgery. The integration of robotic systems into liver surgery promises not only an augmentation of the surgeon's inherent skills but also the potential to perform complex resections with enhanced precision and control. While revolutionary, this technological innovation introduces its own suite of considerations, including significant financial investments, the necessity for specialized training, and ongoing debates regarding its cost‐effectiveness and accessibility. The first guidelines on robotic liver resection were established as early as 2018,^3^ and a revised version was published this year.^4^

This review aims to explore the evolution, current status, and future prospects of robot‐assisted hepatic resection. However, since several review articles have already been published,^5^, ^6^, ^7^ we would like to present a specialized, cutting‐edge review mainly on the latest literature of the past 2 years (2022/23) regarding the following items: “robotic surgery in liver transplantation,” “robotic hepatectomy for hilar cholangiocarcinoma,” “robotic vascular reconstruction following hepatectomy,” “robotic repeat hepatectomy,” and “prospective trials in robotic hepatectomy.”

## 2 ARTICLE RETRIEVAL PROCEDURES IN THIS REVIEW

Using the literature search site PubMed (https://pubmed.ncbi.nlm.nih.gov/), we searched for the above phrases using a 5‐year filter and excluding review literature, with the main focus on those published in 2022/23, and first, the abstracts were reviewed (initial selection). Those whose contents were considered useful in the review of robotic surgery were further reviewed regarding their main texts before being included in this literature review (second selection).

## 3 RESULTS

### 3.1 Robotic surgery in liver transplantation

Of the 11/50 articles that were identified in the initial selection, seven were selected in the second selection. Schulze et al. reported 501 robotic donor liver resections, which is considered one of the largest single‐center cases in Saudi Arabia.^8^ In this report, right lobe grafts accounted for 42% of all grafts, with a median blood loss and operative times of 60 mL and 6.77 h, respectively, open conversion in only two cases (0.4%), and Clavien–Dindo (C‐D) complication grade III or higher in only one case, which was an excellent result. We present two articles that retrospectively compared their results. Kim et al. conducted a retrospective single‐institute cohort study to compare the results of robotic and laparoscopic donor right hemihepatectomies and concluded that robotic surgery resulted in less intraoperative bleeding (104 vs. 238 mL, *p* = 0.002) and comparable postoperative outcomes to laparoscopic surgery.^9^ In a more advanced study, Varghese et al. compared extended donor criteria (graft weight ≥800 g, type 2/3 portal vein, >1 bile duct or hepatic artery, and inferior hepatic veins >5 mm requiring reconstruction) and standard donor criteria in their robotic donor right hepatic resection using propensity score matching, which yielded similar results to those for the standard donor, but the learning curve may require 120 cases, based on their own experience.^10^ Generally, robotic surgery is considered to have a shorter learning curve than laparoscopic surgery; however, its description may be highly self‐critical. The summary of these three articles is shown in Table 1. Regarding robotic donation for pediatric liver transplantation (left lateral segmentectomy), the initial results from Chennai, India, were reported as follows: operative time 327 min, blood loss 50 mL, complications up to C‐D grade IIIA 21.3%, and no open conversion. They also conducted a study using the CUSUM score and showed that the learning curve reached a plateau after 16 cases,^11^ which seems to be in line with clinical findings.

**TABLE 1:** Summary of the latest articles of robotic donor liver resection.

First author	References	Year	Right lobe graft	Operative time	Blood loss	Open conversion	CD; beyond III a
Schulze	^8^	2022	42.0%	6.77	60	2/501 (0.4%)	1/501 (0.2%)
Kim	^9^	2022	100.0%	7.73	104	2/102 (2%)	7/102 (6.8%)
Varghese	^10^	2022	97.8%	9.0^a^	250^a^	1/66 (1.5%)^a^	2/66 (3.3%)^a^

Abbreviations: CD, Clavien–Dindo classification; ref, reference number.

^a^ Indicates the surgical results of the standard anatomy group after propensity score match.

However, we believe that robotic recipient surgery is still in the future, but a team from Seoul, Korea, has already reported a case of robotic recipient surgery. There are several case reports from these studies; therefore, we have cited only one in this review.^12^ They concluded that they would expect robotic recipient surgery to reduce postoperative pain, shorten the length of hospital stay, and improve aesthetics, but we should wait for further reports to determine if surgical safety will also improve.

### 3.2 Robotic hepatectomy for hilar cholangiocarcinoma

Of the 5/16 articles that were identified in the initial selection, seven were selected in the second selection. As might be expected in a field where robotic surgery has not yet penetrated that far, we could not find any large series of publications in this 2‐year period. In case reports, Di Benedetto et al.^13^ and Camerlo et al.^14^ reported robotic left hepatectomy with biliary reconstruction for hilar cholangiocarcinoma with surgical video clips. Not surprisingly, robotic surgery is clearly more stable than laparoscopy for the manipulation of the hilar vessels. In addition, after watching the video clip, we again recognized that the camera was more stable than the laparoscopic video and that the robotic surgery images were very good for video submission. Alternatively, as a distinctive surgery, Di Benedetto et al.^15^ presented a video of a robotic first‐stage procedure in associating liver partition and portal vein ligation for staged hepatectomy surgery. The video shows that the initial operation of the hepatoduodenal ligament can be performed as easily as in open surgery, but the second surgery was performed using open surgery because of adhesions. Deng et al.^16^ reported a video of robotic Taj Mahal surgery. This may be a more suitable technique for robotic hepatectomy because a large liver resection surface may inevitably take longer than laparoscopy.

### 3.3 Robotic vascular reconstruction following hepatectomy

Of the 8/11 articles that were identified in the initial selection, two were selected in the second selection. Magistri et al.^17^ reported their initial 14‐case series of robotic hepatectomy for perihilar cholangiocarcinoma accompanied by a video clip of one case of portal vein reconstruction. In contrast, Wang et al.^18^ reported the first case of robot‐assisted reconstruction of the middle hepatic vein with artificial blood vessels combined with a hepatectomy. Unfortunately, this study did not include a video, so we could not ascertain the details of the surgery; however, according to them, robotic surgery helped to reconstruct the hepatic vein precisely and prevented the occurrence of postoperative complications related to vascular anastomosis and may be feasible in an experienced robotic surgery center.

### 3.4 Robotic repeat hepatectomy

Of the 2/4 articles that were identified in the initial selection, two were selected for the second selection. Machado et al.^19^ reported on the use of robotic repeat hepatectomy (caudate lobe resection) for metastasis from colon cancer after left and partial S8 hepatectomies using a video clip. The authors concluded that the magnification effect facilitated a better structural understanding of the hepatic hilum and that the articulating function was useful for manipulation, even with a narrow field of view. Last year, using their national prospective database, Sandri et al.^20^ reported the usefulness of minimally invasive repeat hepatectomy in a comparison between initial hepatectomy and minimally invasive repeat hepatectomy using propensity score matching, in which robotic repeat hepatectomy was not included. In the future, with the further proliferation and development of robotic surgery, it will be selected more often for repeat hepatectomies.

### 3.5 Prospective clinical trials in robotic hepatectomy

Regarding the most recent paper of the past 2 years, we did not recognize any prospective studies that have demonstrated the benefit of robotic liver resection. Therefore, we reviewed the NIH (ClinicalTrials.gov) website and found only one prospective randomized study on robotic liver resection, titled “Robot‐assisted Procedure Versus Open Simultaneous Resection of Colorectal Cancer with Liver Metastases” by Jianmin et al. (NCT02642978). The results are available online in the form of a European Society for Medical Oncology (ESMO) conference report; however, a full‐size article was not identified in the authors' search. Robotic surgery is also expensive and may require further proof of its usefulness in prospective trials.

In conclusion, we reviewed the most recent articles on robotic liver resection in the last 2 years, especially regarding the most advanced topics. We believe that reports on new resection techniques, especially in the case of robotic surgery, should be accompanied by easy‐to‐view videos that are useful for passing on the techniques. I conclude this article with the hope that this field will continue to develop in the future.

## AUTHOR CONTRIBUTIONS

Conceptualization: TA and HM conceived and designed the review's scope and main objectives. Methodology: TA and TH developed the search methodology and criteria for inclusion and exclusion of studies. Literature search: TA, HM, and TH conducted the literature search and data acquisition. Writing – original draft preparation: TA drafted the manuscript. Writing – review and editing: SA critically revised the work for important intellectual content. Supervision: SE supervised the project and provided guidance throughout the review process.

## FUNDING INFORMATION

No funding was received to conduct this study.

## CONFLICT OF INTEREST STATEMENT

SE is an editorial board member of *Annals of Gastroenterological Surgery*.

## ETHICS STATEMENT

The literature included in this review was selected based on predefined criteria ensuring a comprehensive and unbiased collection of relevant studies. Each article was evaluated for ethical standards and only those with clear ethical approval and adherence to ethical guidelines were included.
